# Disparities and effectiveness of COVID-19 vaccine policies in three representative European countries

**DOI:** 10.1186/s12939-024-02110-w

**Published:** 2024-01-30

**Authors:** Wanzhen Xie, Leiyu Shi, Meiheng Liu, Junyan Yang, Mengyuan Ma, Gang Sun

**Affiliations:** 1 Department of Health Management, School of Health Management, Southern Medical University, Guangzhou, 510515 China; 2 Department of Health Policy and Management, Bloomberg School of Public Health, Johns Hopkins University, Baltimore, MD 21205 USA

**Keywords:** COVID-19, Vaccine equity, Vaccination effectiveness, Vaccine policies disparities, The effective reproduction rate

## Abstract

**Objective:**

The aim of this study was to examine the Coronavirus disease 2019(COVID-19) vaccine policies disparities and effectiveness in Germany, Denmark and Bulgaria, with a view to providing lessons for global vaccination and response to possible outbreak risks.

**Methods:**

This study analyzed big data through public information on the official websites of the Ministries of Health of the European Union, Germany, Denmark and Bulgaria and the official websites of the World Health Organization. We systematically summarized the COVID-19 vaccine policies of the three countries, and selected the following six indicators for cross-cutting vaccination comparisons: COVID-19 vaccine doses administered per 100 people, COVID-19 vaccination rate, the share of people with fully vaccinated, the share of people only partly vaccinated, cumulative confirmed COVID-19 cases per million, cumulative confirmed COVID-19 deaths per million. Meanwhile, we selected the following four indicators for measuring the effectiveness of COVID-19 vaccine policy implementation: daily cases per million, daily deaths per million, the effective reproduction rate (Rt), the moving-average case fatality rate (CFR).

**Results:**

Although these three EU countries had the same start time for vaccination, and the COVID-19 vaccine supply was coordinated by the EU, there are still differences in vaccination priorities, vaccination types, and vaccine appointment methods. Compared to Germany and Denmark, Bulgaria had the least efficient vaccination efforts and the worst vaccination coverage, with a vaccination rate of just over 30% as of June 2023, and the maximum daily deaths per million since vaccination began in the country was more than three times that of the other two countries. From the perspective of implementation effect, vaccination has a certain effect on reducing infection rate and death rate, but the spread of new mutant strains obviously aggravates the severity of the epidemic and reduces the effectiveness of the vaccine. Among them, the spread of the Omicron mutant strain had the most serious impact on the three countries, showing an obvious epidemic peak.

**Conclusions:**

Expanding vaccination coverage has played a positive role in reducing COVID-19 infection and mortality rates and stabilizing Rt. Priority vaccination strategies targeting older people and at-risk groups have been shown to be effective in reducing COVID-19 case severity and mortality in the population. However, the emergence and spread of new variant strains, and the relaxation of epidemic prevention policies, still led to multiple outbreaks peaking. In addition, vaccine hesitancy, mistrust in government and ill-prepared health systems are hampering vaccination efforts. Among the notable ones are divergent types of responses to vaccine safety issue could fuel mistrust and hesitancy around vaccination. At this stage, it is also necessary to continue to include COVID-19 vaccination in priority vaccination plans and promote booster vaccination to prevent severe illness and death. Improving the fairness of vaccine distribution and reducing the degree of vaccine hesitancy are the focus of future vaccination work.

## Introduction

The SARS-CoV-2 virus and the COVID-19 disease that it caused triggered the most serious public health emergency in more than a century [1]. On 31 January 2020, the World Health Organization (WHO) declared that COVID-19 constituted a global "pandemic" and classified it as a public health emergency of international concern. Based on the epidemic situation, the characteristics of virus mutation, the immune barrier of the population and the building of response capacity, WHO declared that COVID-19 no longer constitutes a "public health emergency of international concern" on 5 May 2023.

As of July 10, 2023, COVID-19 has caused more than 700 million confirmed cases and more than 6 million deaths worldwide [2]. In order to combat the epidemic, countries have actively developed COVID-19 prevention and vaccination policies. Vaccines in EU countries are uniformly reviewed by the European Medicines Agency (EMA), which authorizes vaccines from different manufacturers after a positive assessment of vaccine safety and efficacy [3]. The European Commission, on behalf of Member States, will enter into pre-purchase agreements with individual vaccine manufacturers in order for member States to have access to vaccines in the fight against the COVID-19 pandemic, with the principle of distribution of vaccines among Member States on a population-based basis, ensuring equitable distribution of vaccines among Member States on a population-based basis [4]. At present, EMA has authorized a total of eight COVID-19 vaccines for use in the EU, developed and manufactured by the companies Pfizer & BioNTech, AstraZeneca, Johnson & Johnson, Moderna, Novavax, HIPRA, Sanofi Pasteur, and Valneva [5]. While vaccines in EU countries are centrally coordinated, the COVID-19 pandemic has exposed the fragmented nature of government policy decisions implemented in Europe and the differences in effectiveness. Since the EU is not permitted to interfere with national health systems and can only ‘‘support Member States in their endeavors to combat cross-country health scourges” (Article 168(5) in the Treaty on the Functioning of the European Union), European countries can develop independent national vaccination policies [6].

Three EU countries, Germany, Denmark and Bulgaria, were selected in this study for the following reasons: (1) There are differences in the vaccination rates of the three countries. The vaccination rates of Germany and Denmark are at the forefront of EU countries, while the vaccination rates of Bulgaria are at the bottom of EU countries. (2) The EU intervenes in the vaccine deployment process in the three countries, which are both EU countries, but there are differences in vaccination and epidemic control [3]. (3) These three countries are representative of the EU countries in terms of vaccination, epidemic control and economy. Among them, Germany is the largest national economy in the European Union with a large population, with the first pharmaceutical research and development company that obtained the EU conditional marketing authorization for BNT162 COVID-19 vaccine, and is one of the COVID-19 vaccine production bases approved by the European Medicines Agency (EMA), supplying a large number of COVID-19 vaccines for domestic, other EU member states and even the world. Denmark, a relatively small country with a developed economy, has demonstrated a good vaccination process, being the first country in the EU to announce the lifting of all vaccination restrictions and the first country in the world to halt its COVID-19 vaccination program. Bulgaria has the smallest population density compared to the other two countries, is one of the less developed countries in the EU, and has one of the highest COVID-19 mortality rates in the EU. In addition, the three countries, as different types of countries in Europe, can help to understand the implementation and effects of policies in different national contexts.

The aim of this study was to examine the Coronavirus disease 2019(COVID-19) vaccine policies disparities and effectiveness in Germany, Denmark and Bulgaria, with a view to identifying the factors that bring about an impact on vaccination, and to provide lessons for advancing the process of global vaccination efforts and coping with the risks of possible epidemics.

## Materials and methods

### Data collection

The COVID-19 epidemiological data used in this study were obtained from Our World in Data Website, the official websites of the national ministries of health of Germany, Denmark and Bulgaria. The data were selected for the period from the start of COVID-19 vaccination in each of the three countries until 1 June 2023 (Data for Figs. 2, 3 and 4 were collected only until December 27, 2022 for national statistical reasons.). COVID-19 vaccine doses administered per 100 people, COVID-19 vaccination rate, share of people with fully vaccinated, share of people only partly vaccinated, cumulative confirmed cases per million, cumulative confirmed COVID-19 deaths per million were selected for cross-sectional comparison of COVID-19 vaccination in these three countries. Daily cases per million, daily deaths per million, the effective reproduction rate (Rt), the moving-average case fatality rate (CFR) were calculated to measure the effectiveness of COVID-19 vaccine policies implementation in each of these three countries. Table 1 provides a detailed definition of the indicators.

**Table 1:** Definition of each indicator

Indicator	Definition
COVID-19 vaccine doses administered per 100 people	Total number of COVID-19 vaccinations received per 100 people in the country's total population
COVID-19 vaccination rate	The proportion of the country's total population who have received at least one dose of COVID-19 vaccine, including the proportion of the population who have been fully vaccinated and the proportion of the population who have been partially vaccinated
share of people with fully vaccinated	The total number of people who received all doses prescribed by the initial vaccination protocol (e.g. 2 doses for Pfizer/BioNTech, Moderna, Oxford/AstraZeneca, etc. and 1 dose for Johnson & Johnson), divided by the country's total population
share of people only partly vaccinated	The total number of people who have received only their first vaccine dose, divided by the country's total population
cumulative confirmed cases per million	The cumulative total of confirmed COVID-19 cases per 1 million people in the country. The count can include reported possible cases
cumulative confirmed COVID-19 deaths per million	The cumulative total COVID-19 deaths per 1 million people in the country. The count may include reported possible deaths
daily cases per million	The total number of COVID-19 cases diagnosed per 1 million people per day in the country. The count can include reported possible cases
daily deaths per million	The total number of COVID-19 deaths per 1 million people per day in the country. The count may include reported possible deaths
the effective reproduction rate (Rt)	The reproduction rate represents the average number of new infections caused by a single infected individual. If the rate is greater than 1, the infection is able to spread in the population. If it is below 1, the number of cases occurring in the population will gradually decrease to zero
The moving-average case fatality rate (CFR)	The case fatality rate (CFR) is the ratio between confirmed deaths and confirmed cases. The moving-average CFR is calculated as the ratio between the 7-day average number of deaths and the 7-day average number of cases 10 days earlier

The effective reproduction rate (Rt) is an indicator of how many people are infected by one individual in the process of disease transmission and development [7]. If Rt = 2, this means that each infected person spreads the virus to two other people, leading to an uncontrolled epidemic. Achieving Rt < 1 is an important condition for preventing virus transmission [8]. Therefore, this indicator can be selected to assess the impact of vaccine policy based on the severity of COVID-19 pandemic at the time. The case fatality rate (CFR) is the ratio between confirmed deaths and confirmed cases. The moving-average case fatality rate was used to evaluate and measure the effectiveness of vaccine policy and it was calculated as the ratio between the 7-day average number of deaths and the 7-day average number of cases 10 days earlier. It can directly reflect the severity of the epidemic and be used to compare the effects of different regions, different time periods or different vaccine policies.

## Results

### Basic information on COVID-19 vaccination in the three countries

As can be seen from Table 2, the basic COVID-19 vaccination situation in Germany, Denmark and Bulgaria has some similarities, but also some differences. Due to EU-mediated intervention during the vaccine deployment process [9], vaccination started at the same time in all three countries and received COVID-19 vaccines that had received conditional marketing authorizations from the European Commission. Among them, Denmark announced the discontinuation of AstraZeneca and Johnson & Johnson vaccines due to the safety concerns about rare thrombosis associated with the vaccine [10].

**Table 2:** Basic information on COVID-19 vaccination in Germany, Denmark and Bulgaria

	Germany	Denmark	Bulgaria
Start date of vaccination	December 27, 2020	December 27, 2020	December 27, 2020
Vaccines administered	Pfizer/BioNTech, Moderna, Novavax, Valneva, Sanofi Pasteur, Johnson & Johnson, AstraZeneca	Pfizer/BioNTech, Moderna	Pfizer/BioNTech, Moderna, AstraZeneca, Johnson & Johnson
COVID-19 vaccine doses administered per 100 people	230.56	254.54	67.99
COVID-19 vaccination rate	77.9%	81.2%	31.07%
Share of people with fully vaccinated	76. 4%	80.6%	30.62%
Share of people only partly vaccinated	1.50%	0.60%	0.45%
Share of people with booster vaccinated	62.6%	61.86%	13.83%
Vaccines Appointment	Vaccination appointments can be made online and over the phone, as well as through GP surgeries, paediatric clinics, many other specialist clinics that offer vaccinations against COVID-19, company doctors and some pharmacies that also offer vaccinations against COVID-19	If you are offered vaccination based on your age, you will receive an invitation for vaccination. Instead, you can book an appointment at a vaccination center www.vacciner.dk using your NemID/MitID	Vaccine appointments are made online by all people from April 19, 2021 to July 1, 2022 Besides, COVID-19 vaccinations can be administered by contacting their GP, local health inspectorates, mobile vaccination sites and medical facilities
Free vaccination or not	Free vaccinations for the entire population	Free vaccinations for the entire population in the early stage. From 15 November 2022, Danish citizens who are not in the at-risk group will be required to pay for the COVID-19 vaccine	Free vaccination for the entire population

The source of the data is the official websites of the national ministries of health of the three countries and https://ourworldindata.org/covid-vaccinations (accessed on 1 June 2023)

There is basically no big difference in the way of COVID-19 vaccine appointment in these three countries, and all of them can make an appointment for vaccination through the online appointment system, and all of them have realized free vaccination. Germany made vaccine appointment and vaccination according to the priority order of vaccination in the early stage. With the improvement of vaccination rate and gradually sufficient vaccine supply, the priority order of vaccination was cancelled in the later stage, and everyone has the right to make an appointment for vaccination. In Denmark, vaccination invitations are issued according to age. Citizens who are not in the specified age group need to make an appointment by themselves. In order to improve the vaccination rate, free vaccination is provided in the early stage, and those who are not at risk of COVID-19 virus infection need to pay for the vaccination at their own expense in the later stage. In Bulgaria, due to the low willingness of citizens to vaccinate, the online reservation system stopped functioning at a later stage, and no appointment was required for vaccinations.

**Fig. 1:**
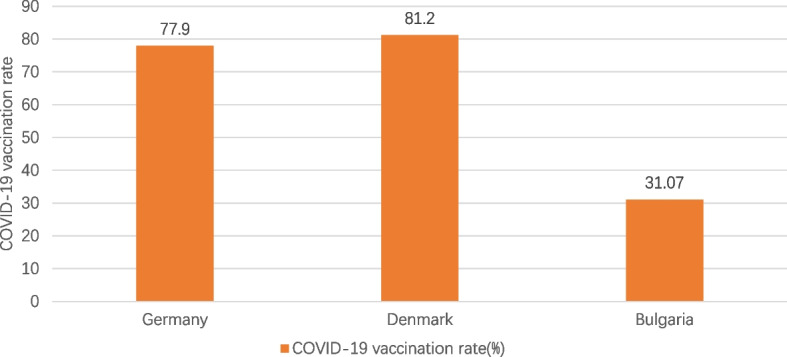
COVID-19 vaccination rate in the three countries. Note1: The data were selected for the period from the start of COVID-19 vaccination in each of the three countries until 1 June 2023. Note2: The source of the data is the official websites of the national ministries of health of Germany, Denmark and Bulgaria. The data source for Germany is https://impfdashboard.de/en/ (accessed on 1 June 2023). The data source of Denmark is https://www.sst.dk/en/english/corona-eng/vaccination-against-covid-19 (accessed on 1 June 2023). The data source of Bulgaria is https://coronavirus.bg/bg/statistika (accessed on 1 June 2023)

**Fig. 2:**
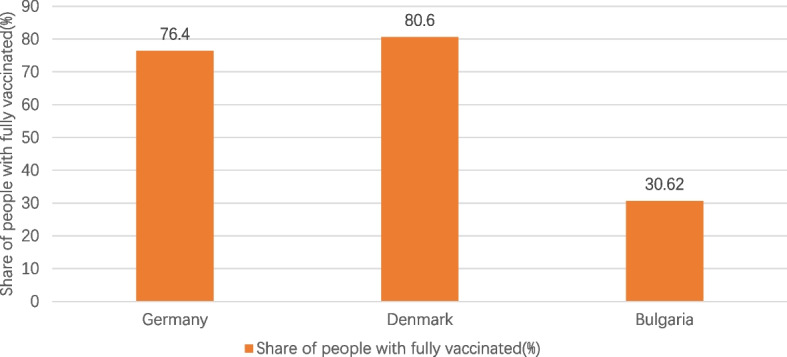
Share of people with fully vaccinated in the three countries. Note1: The data were selected for the period from the start of COVID-19 vaccination in each of the three countries until 1 June 2023. Note2: The source of the data is the official websites of the national ministries of health of Germany, Denmark and Bulgaria. The data source for Germany is https://impfdashboard.de/en/ (accessed on 1 June 2023). The data source of Denmark is https://www.sst.dk/en/english/corona-eng/vaccination-against-covid-19 (accessed on 1 June 2023). The data source of Bulgaria is https://coronavirus.bg/bg/statistika (accessed on 1 June 2023)

**Fig. 3:**
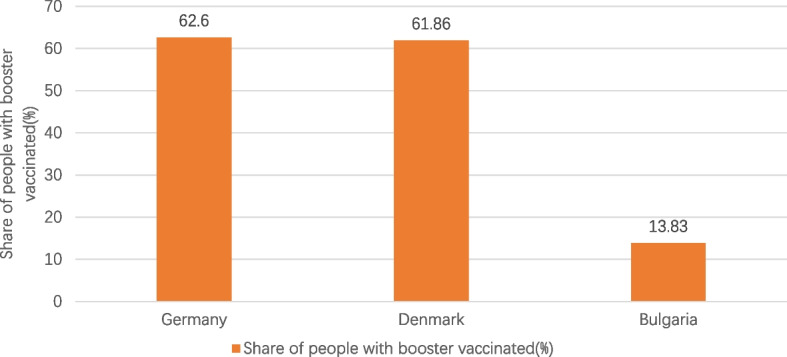
Share of people with booster vaccinated in the three countries. Note1: The data were selected for the period from the start of COVID-19 vaccination in each of the three countries until 1 June 2023. Note2: The source of the data is the official websites of the national ministries of health of Germany, Denmark and Bulgaria. The data source for Germany is https://impfdashboard.de/en/ (accessed on 1 June 2023). The data source of Denmark is https://www.sst.dk/en/english/corona-eng/vaccination-against-covid-19 (accessed on 1 June 2023). The data source of Bulgaria is https://coronavirus.bg/bg/statistika (accessed on 1 June 2023)

**Fig. 4:**
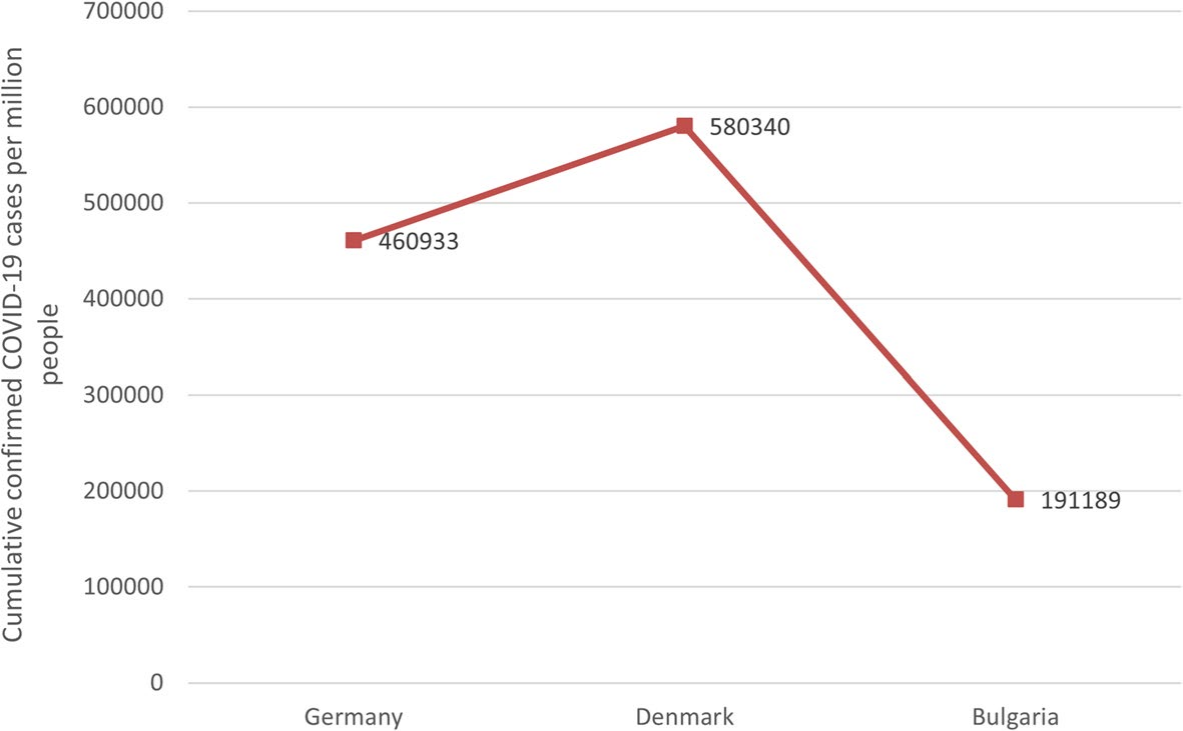
Cumulative confirmed COVID-19 cases per million people in the three countries. Note1: The data were selected for the period from the start of COVID-19 vaccination in each of the three countries until 1 June 2023. Note2: The source of the data is https://ourworldindata.org/covid-vaccinations (accessed on 1 June 2023)

**Fig. 5:**
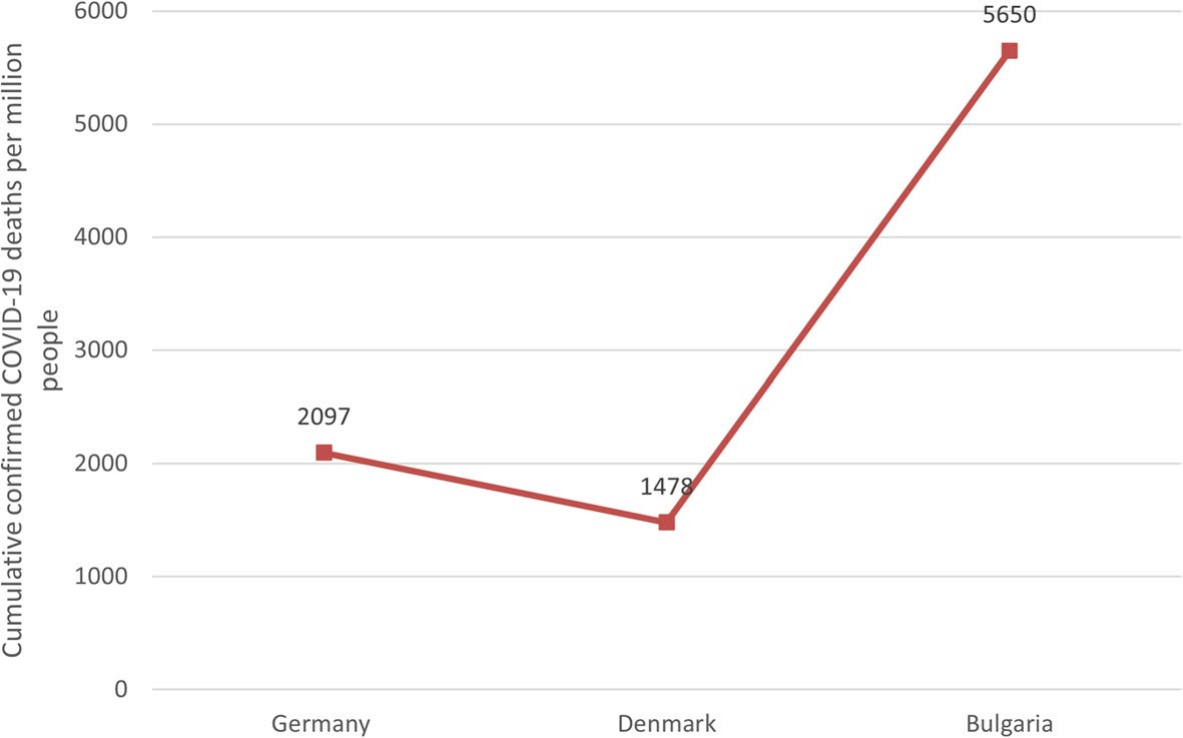
Cumulative confirmed COVID-19 deaths per million people in the three countries. Note1: The data were selected for the period from the start of COVID-19 vaccination in each of the three countries until 1 June 2023. Note2: The source of the data is https://ourworldindata.org/covid-vaccinations (accessed on 1 June 2023)

It can be seen in Figs. 1, 2, 3, 4 and 5 that there is a small difference in the coverage of COVID-19 vaccination between Germany and Denmark. Although the proportion of the population with vaccine boosters is lower in Denmark than in Germany, the total vaccination rate and the proportion of the population fully vaccinated are the highest among these three countries. Being also a member of the European Union, with vaccine supply guaranteed by the European Commission, Bulgaria has the lowest level of vaccination compared to the other two countries, with the lowest level of all indicators. In addition, the cumulative confirmed COVID-19 cases per million people in the three countries were significantly different, with Denmark having the most cases and Bulgaria having the least. Cumulative confirmed COVID-19 deaths per million people, by contrast, Bulgaria has the most and Denmark has the least.

### Core COVID-19 vaccine policies of the three countries

#### Germany

The German COVID-19 vaccine policies can be found in Table 3. Germany launched mass COVID-19 vaccination on 27 December 2020, and as of June 2023, vaccination coverage had reached 77.9%. Germany had kept mortality rates low throughout the first wave of the European epidemic, but due to insufficient vaccine quantities, and as one of the most aging countries in the EU, Germany had set up an almost draconian program for vaccination. German Standing Committee on Vaccination (STIKO) had recommended six priority levels for the vaccination sequence, based on the likelihood of the population developing severe symptoms and the degree of contact with people at risk or infected. At the same time, the legally binding vaccination regulations divide the population into four priority groups, ensuring the vaccination of the elderly, workers in special positions and other at-risk groups to reduce the damage of the virus to human health. On 7 June 2021, the priority order for vaccination was abolished and the whole population to COVID-19 vaccination was introduced.

**Table 3:** COVID-19 vaccine policies of Germany

Aspects	Policies
Vaccination sequence (Four priority groups)	1. **Highest priority:** people aged 80 years and above; Persons receiving treatment, care or work in inpatient facilities for the elderly or persons in need of care; Nursing staff in outpatient care services; Employees of medical institutions; Employees of medical facilities that treat, care for, or provide care to at-risk populations 2. **High priority:** people over 70 years old; People with trisomy 21 syndrome, dementia or mental retardation after organ transplantation; Persons in close contact with persons over 80 years of age or residents of nursing homes and homes for the mentally handicapped; Close contacts with pregnant women; A person who works in an inpatient facility for a mentally handicapped person or who regularly treats, cares for or cares for a mentally handicapped person in an outpatient care service; People who work in healthcare facilities with a high or increased risk of coronavirus exposure, especially doctors and other personnel who have frequent contact with patients, blood and plasma service personnel, and personnel in testing centers; Police and law enforcement personnel are at high risk of infection while on duty (e.g. during demonstrations); People in positions related to public health services and hospital infrastructure; Individuals living or working in refugee and homeless facilities 3. **Higher priority:** people over 60 years old; Individuals with the following conditions: obesity, chronic kidney disease, chronic liver disease, immune deficiency or HIV infection, diabetes, heart disease, stroke, cancer, chronic obstructive pulmonary disease or asthma, autoimmune diseases and rheumatism; Exposure to low-risk health care facilities (laboratories) and employees who do not care for patients with suspected infectious diseases; Persons holding relevant positions in government, executive and constitutional bodies, armed forces, police, fire brigade, disaster control and THW, judiciary; Key infrastructure companies, pharmacy and pharmaceutical industry, public supply and disposal, food industry, transportation, information technology and telecommunications; Educators and teachers; A person whose work or living conditions are precarious 4.** Other people**
Vaccine development and supply	1. In mid-November 2020, BioNTech of Germany and Pfizer of the United States successfully developed the COVID-19 vaccine (Comirnaty) 2. On December 21, 2020, EMA recommended granting a conditional marketing authorization for the vaccine Comirnaty, developed by BioNTech and Pfizer 3. On 31 December 2020, Comirnaty received an emergency use authorization from the WHO 4. In August 2021, Comirnaty received official FDA approval (for people over 16 years of age), becoming the first fully marketed COVID-19 vaccine 5. Since August 2021, Germany had committed to support COVAX, the global vaccination initiative. In 2021 and 2022, around 136 million vaccine doses were transferred to COVAX. In addition, the Federal Government bilaterally donated approximately 10 million doses to six different countries(Egypt, Ghana, Namibia, Thailand, Ukraine and Viet Nam). This means over 146 million doses were donated in total 6. On 2 September 2022, the European Commission approved the improved booster vaccine Comirnaty Original/Omicron Ba.1 7. As of 15 September 2022, the European Commission has authorized the use of the Comirnaty Original/ Omicron BA.4–5 modified booster vaccine 8. On October 10, 2022, the European Commission converted Comirnaty from a conditional marketing authorization to a standard marketing authorization
Vaccination of minors	1. On June 7, 2021, German federal and state governments agreed to start offering COVID-19 vaccination to children over the age of 12 2. On December 13, 2021, Germany began vaccinating children between the ages of 5 and 11 against COVID-19 3. In mid-November 2022, the German Standing Committee on Vaccination (STIKO) said that young children at increased risk of severe COVID-19 due to pre-existing conditions should be vaccinated, and the recommendation applies to children between six months and four years of age 4. In late April 2023, the German Standing Committee on Vaccination (STIKO) will no longer recommend that healthy children and adolescents in Germany be vaccinated against COVID-19 in view of the waning of the pandemic. Children who are in the risk group for severe Covid-19 due to illness are still recommended to be vaccinated starting at six months of age
Compulsory vaccination	No compulsory COVID-19 vaccination
Vaccination boosters	1. In September 2021, Germany began offering the first booster dose of the COVID-19 vaccine to the elderly and people with weakened immune systems 2. In October 2021, the German Standing Committee on Vaccination (STIKO) recommended the booster vaccination against COVID-19 for people over 70 years of age and for certain indications, as well as additional doses of mRNA vaccination for people receiving the COVID-19 vaccine Janssen. It is recommended that the third dose should be given 6 months after the formation of basic immunity 3. On 18 November 2021, the German Standing Committee on Vaccination (STIKO) recommended that all people aged 18 and over receive an mRNA booster vaccination with the third dose to be given six months after basic immunity has developed 4. At the end of December 2021, the German Standing Committee on Vaccination (STIKO) recommended that adults be given the booster vaccination after 3 months of full vaccination (previously it was recommended after 6 months) 5. In January 2022, the German Standing Committee on Vaccination (STIKO) recommended the first dose of booster vaccination for all children and adolescents aged 12–17 years 6. In mid-February 2022, the German Standing Committee on Vaccination (STIKO) recommended that people over 70 years of age, residents and caregivers of nursing facilities, persons over 5 years of age with immune deficiencies, and staff of medical and nursing facilities (especially those in direct contact with patients and residents) receive a second booster dose, at the earliest three months after the first booster dose. For people working in healthcare and nursing facilities, it is recommended to get the vaccine 6 months after the first booster dose of the vaccine 7. In mid-August 2022, the German Standing Committee on Vaccination (STIKO) recommended that people aged 60–69 years and those over 5 years old who are at risk of severe COVID-19 due to underlying diseases should receive a second booster dose of COVID-19 vaccine
Incentivize vaccination	From September 13 to September 19, 2021, Germany will start a nationwide "Vaccination Week" (Impfaktionswoche), in which people can voluntarily receive free COVID-19 vaccinations without prior appointment at any place marked "Hier wird geimpft" (Vaccination available here), including buses, libraries, football stadiums and shopping malls
Vaccination support policies	1. From mid-June 2021, the German Digital Vaccination Certificate was put into use, and doctors' clinics, vaccination centers and pharmacies were able to provide QR code digital authentication for vaccinated people. People can prove they have been vaccinated simply through the CovPass or Corona-Warning-app mobile App. The certificate showed whether the holder had been vaccinated and how long it had been vaccinated, and can be used in place of a negative test certificate in situations such as travel 2. EU Digital Covid Certificates, also known as "Green digital Certificates", was officially launched on July 1, applicable to the 27 EU Member States (excluding Ireland) and four non-EU countries: Iceland, Norway, Switzerland, and Liechtenstein

The sources of vaccine policy details are https://www.rki.de/DE/Content/Infekt/EpidBull/Archiv/2021/Ausgaben/02_21.pdf;jsessionid=6E8DBF63A782804B070AAAC1C47719E8.internet061?__blob=publicationFile *(accessed on* 1 June 2023*).* https://www.bundesgesundheitsministerium.de/en/*(accessed on* 1 June 2023*).* https://www.infektionsschutz.de/coronavirus/schutzimpfung/*(accessed on* 1 June 2023*).* https://www.rki.de/DE/Home/homepage_node.html*(accessed on* 1 June 2023*).* https://www.zeit.de/index*(accessed on* 1 June 2023*)*

BioNTech is a German biopharmaceutical company that has developed the first fully marketed COVID-19 vaccine, the Comirnaty vaccine, in collaboration with Pfizer, USA. As one of the COVID-19 vaccine production sites approved by the European Medicines Agency (EMA), Germany supplied a large number of COVID-19 vaccines to the country, other EU member states and the world. In addition, as demand for vaccines declines, Germany has a surplus of vaccines. As a result, Germany had donated a total of more than 146 million doses of COVID-19 vaccine to COVAX through the global vaccination initiative, contributing significantly to promoting Equitable Global Distribution of COVID-19 Vaccine.

In the case of oversupply of vaccines and insufficient willingness of people to vaccinate, in addition to launching a national "vaccine week" [11, 12], Germany has also held creative measures such as "vaccination night" musical parties and free food to motivate people to vaccinate against COVID-19 in order to protect people from serious complications of the novel coronavirus [13, 14].

#### Denmark

The COVID-19 vaccine policies of Denmark can be found in Table 4. According to the vaccination calendar published by the Danish Health Authority, the target population at each stage would receive an invitation to be vaccinated against COVID-19. During the vaccine shortage phase, Denmark prioritized high-risk groups and workers in special positions, but the vaccination process lagged behind the vaccination calendar. With the gradual easing of the shortage of vaccine supply in the European Union, the Danish vaccination population had gradually expanded and began to arrange vaccine invitations and vaccinations according to age. Denmark's vaccination rate has been at the forefront of the EU countries and is one of the countries with the highest vaccination rate in the world, reaching 81.2% by June 2023.

**Table 4:** COVID-19 vaccine policies of Denmark

Aspects	Policies
Basic vaccination schedule	1. **Phase I vaccination**: nursing home residents, individuals aged 85 years or older, vulnerable and frontline personnel 2. **Phase II vaccination**: the age group above 16 years old were vaccinated in descending order
Vaccine research and development	1. In February 2021, the Danish Medicines Agency collaborated with national medicines authorities in the EU/EEA, the European Medicines Agency (EMA) and the World Health Organization (WHO) to monitor the safety of COVID-19 vaccines 2. In March 2021, the vaccine "ABNCoV2", developed by a research team at the University of Copenhagen in collaboration with AdaptVac, ExpreS2ion Biotechnologies and Bavarian Nordic, was approved for clinical trials
Vaccination of minors	1. On 27 December 2020, Denmark began vaccinating people over the age of 16 against COVID-19 2. On 17 June 2021, the Danish Health Authority decided to recommend vaccination for children aged 12–15 years 3. In November 2021, children aged 5–11 years in Denmark were included in the vaccination program 4. From 1 July 2022, children and adolescents under the age of 18 were no longer offered the first dose of COVID-19 vaccine in Denmark, and from 1 September 2022, they were no longer able to receive a second dose
Compulsory vaccination	No compulsory COVID-19 vaccination
Vaccination boosters	1. At the end of September 2021, the Danish Health Authority proposed the first booster dose of the COVID-19 vaccination program, with the first phase covering residents of nursing homes and people with particularly weak immune systems, those aged 85 years and above 2. On October 15, 2021, the second phase began and the first booster dose of the COVID-19 vaccine are offered to individuals aged 65–84 years, people at a particularly increased risk of running a serious COVID-19 course and healthcare and elderly service staff。 3. In November 2021, the Danish Health Authority recommended that everyone over the age of 18 receive the first booster vaccine 4. In mid-January 2022, the second booster dose of the vaccine started to be given to high-risk groups in Denmark, especially those diagnosed with serious diseases 5. On 18 February 2022, the Danish Health Authority assessed that people under 18 years of age should not receive the third dose of vaccine 6. On September 15, 2022, Denmark's fourth COVID-19 vaccination program began, nursing home residents and people aged 85 and over started to be offered vaccination 7. On October 1, 2022, the fourth dose of COVID-19 vaccine will be fully promoted among people aged 50 and above in Denmark
Vaccination support policies	1. From 6 April 2021, Denmark announced a trial of the Corona pass. Became the first country in Europe to introduce digital COVID-19 vaccine credentials. People were required to show a "coronavirus pass" to enter public places and follow quarantine regulations such as wearing masks and keeping a safe distance 2. EU Digital Covid Certificates, also known as "Green digital Certificates", was officially launched on July 1, applicable to the 27 EU Member States (excluding Ireland) and four non-EU countries: Iceland, Norway, Switzerland and Liechtenstein

The sources of vaccine policy details are https://www.sst.dk/en/english (accessed on 1 June 2023). https://laegemiddelstyrelsen.dk/da/nyheder/ (accessed on 1 June 2023)

Public trust and cooperation provided the basis for Denmark's vaccination efforts [15, 16]. As vaccination rates increased and hospital admissions declined steadily, Denmark suspended the general COVID-19 vaccination program on May 15, 2022. Vaccination invitations were no longer being issued, but people could still go to vaccination sites across the country that were still open. By mid-September 2022, as the number of COVID-19 infections increased in the fall, the fourth dose of COVID-19 vaccination for vulnerable populations began. In general, Denmark had implemented vaccination programs according to the development of the epidemic.

#### Bulgaria

The COVID-19 vaccine policies of Bulgaria can be found in Table 5. Like most EU countries, Bulgaria started its COVID-19 vaccination program on December 27, 2020. Since the start of the vaccination program, Bulgaria has ranked one of the lowest vaccination rates in the EU due to vaccine shortages, high public distrust of vaccines, and confusion in the vaccination process [17], 24. While most EU countries almost prioritized the elderly for vaccination, Bulgaria's vaccination program still put the elderly in the second tier despite the country's aging problem, giving priority to vaccinating young people who were at low risk of infection and in their prime. The Bulgarian government had started to expand the vaccination population after the initial vaccination program resulted in a catastrophic high death rate from COVID-19, but low public willingness to vaccinate was still hampering the country's vaccination process. As a result, Bulgaria had taken a number of measures to promote and incentivize vaccination, such as cash incentives, the " + me" campaign, etc., to protect people from serious complications of COVID-19. Even though Bulgaria was actively promoting domestic vaccination through various means, the growth rate of COVID-19 vaccination in Bulgaria had been relatively slow, far below the world average.

**Table 5:** COVID-19 vaccine policies of Bulgaria

Aspects	Policies
Basic vaccination schedule	1. **Phase I vaccination**: doctors, nurses and medical assistants 2. **Phase II vaccination**: users and employees of social institutions, teachers and employees of mink farms 3. **Phase III vaccination**: national infrastructure maintenance work, such as employees of the water supply sector 4. **Phase IV vaccination**: people over 65 years old and those with concomitant disease 5. **Phase V vaccination**: vulnerable populations whose lifestyles are highly susceptible to the epidemic
Expand the vaccination population	1. In mid-February 2021, Bulgaria opened the "green corridors". Regardless of priorities, all adults can be vaccinated 2. On May 17, 2021, the Bulgarian Ministry of Health instructed general practitioners and vaccination centres to vaccinate people over 60 years of age and those with serious chronic diseases 3. On 28 May 2021, the Bulgarian Ministry of Health instructed general practitioners to vaccinate persons outside the scope of their patient lists and those who wish to be vaccinated
Vaccination of minors	As of June 3, 2021, children over 12 years of age in Bulgaria can be vaccinated against COVID-19
Compulsory vaccination	No compulsory COVID-19 vaccination
Vaccine procurement and supply	1. As of 1 December 2022, Bulgaria had donated 172,500 doses of Vaxzevria vaccine to the Kingdom of Bhutan, 50,000 doses of Vaxzevria vaccine to Bosnia and Herzegovina and 51,480 doses of Comirnaty vaccine to the Republic of North Macedonia. 270,000 doses of Vaxzevria vaccine were donated to Bangladesh, 258,570 doses of Comirnaty vaccine to Bosnia and Herzegovina, and 2,830,400 doses of Vaxzevria vaccine to the Islamic Republic of Iran 2. Bulgaria sold £100,000 of the Modena vaccine to the Kingdom of Norway 3. On 11 April 2023, Bulgaria and Poland sent a common position to the President of the European Commission on the contract for the purchase of COVID-19 vaccines: not to join the renegotiation of new vaccine supplies, as we know in advance that these vaccines will be cancelled, asking our countries to decide for themselves the quantity of vaccines we will purchase and pay for them after delivery
Vaccination support policies	1. EU Digital Covid Certificates, also known as "Green digital Certificates", was officially launched on July 1, applicable to the 27 EU member States (excluding Ireland) and four non-EU countries: Iceland, Norway, Switzerland and Liechtenstein 2. On 21 October 2021, green certificates for COVID-19 vaccination, survival or negative test results, which are required for visits to cinemas, theatres, museums, galleries, food and entertainment venues, fitness and gymnasiums, etc., came into use in Bulgaria 3. On 10 March 2022, visitors to sites of public interest should not present a "green certificate" in Bulgaria 4. As of 21 March 2022, all requirements for green certificates in Bulgaria were abolished
Incentivize vaccination	1. On September 30, 2021, the Bulgarian Ministry of Health held a vaccine incentive game to promote the vaccination of COVID-19 in the country, and the winner would be decided randomly by drawing lots 2. On December 23, 2021, the Prime Minister of Bulgaria announced that in order to increase the vaccination rate of the elderly population, the government would give cash incentives to the elderly who received COVID-19 vaccines, and the government stipulates: Every retired Bulgarian who receives the first or second dose of the vaccine will receive a one-time cash payment of 75 Bulgarian levs in addition to the pension for the following six months. Retirees who have already received their third booster dose will also be eligible for the bonus 3. On 21 March 2022, Bulgaria launched the " + Me" campaign to disseminate scientific information on COVID-19 vaccines and the benefits of vaccination
Vaccination boosters	1. On 23 September 2021, the Bulgarian Ministry of Health and the Advisory Committee of Experts on the Supervision of Immunization and Prophylaxis recommended that immunically impaired persons, persons living in nursing homes and residential social institutions, health workers at high risk of infection, persons over 65 years of age, persons who had not built up sufficient immunity one month after the end of the vaccination cycle receive a booster dose of COVID-19 vaccine. Booster doses should be given at the earliest after 6 months 2. On 31 December 2021, the Bulgarian Ministry of Health and the Advisory Committee of Experts on Surveillance of Immunization recommended that the booster be administered no earlier than 3 months after completion of primary vaccination 3. On 25 May 2022, children aged 12–17 years in Bulgaria could receive the Comirnaty booster vaccine at least 3 months after completing the primary vaccination process.。 4. On 24 June 2022, the Bulgarian Ministry of Health approved the recommendation of the Expert Committee on the Surveillance of Immunology and Prophylaxis on the application of the fourth vaccine to all people aged 18 years and above for the prevention of COVID-19, with the second booster dose being administered at least 4 months after the first booster dose 5. In August 2022, the Bulgarian Ministry of Health and the Expert Committee for the Surveillance of Immunization and Prophylaxis recommended that a second booster vaccine against COVID-19 be administered to people over 18 years of age who had received the Janssen vaccine primary immunization

The sources of vaccine policy details are https://www.mh.government.bg/bg/covid-19/informaciya-otnosno-noviya-koronavirus-2019-ncov/*(accessed on* 1 June 2023*).* https://plusmen.bg/*(accessed on* 1 June 2023*)*

Due to the low willingness of the Bulgarian people to vaccinate, a large number of vaccines purchased and supplied by the EU are in surplus, and Bulgaria had donated its surplus COVID-19 vaccine to several countries by December 2022.

### The effectiveness of COVID-19 vaccination in these three countries

**Fig. 6:**
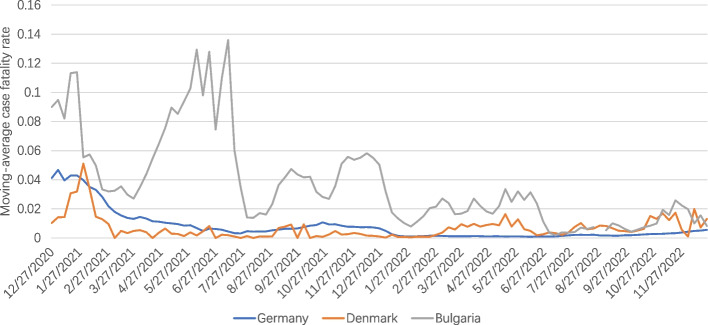
Curves of moving-average case fatality rate of COVID-19. Note1: The case fatality rate (CFR) is the ratio between confirmed deaths and confirmed cases. The moving-average CFR is calculated as the ratio between the 7-day average number of deaths and the 7-day average number of cases 10 days earlier. Note2: The data were selected for the period from the start of COVID-19 vaccination in each of the three countries until 25 December 2023. Note3: The source of the data is https://ourworldindata.org/covid-vaccinations (accessed on 31 December 2023)

Looking at Fig. 6, we can see that the moving-average case fatality rate of COVID-19 in all three countries was relatively high in the early stage of the vaccination program, and then gradually leveled off in the later stage. This can be attributed to a number of factors, including the gradual increase in vaccination coverage in all three countries, the decreased pathogenicity of Omicron, and the improvement in detection capacity in each country. In terms of country differences, Bulgaria had the highest moving-average case fatality rate, which may explain the lower vaccination coverage in that country. Certainly, some studies have found that CFR was much lower in high-income countries due to the fact that high-income countries conducted more COVID-19 testing than low-income countries.

#### Germany

**Fig. 7:**
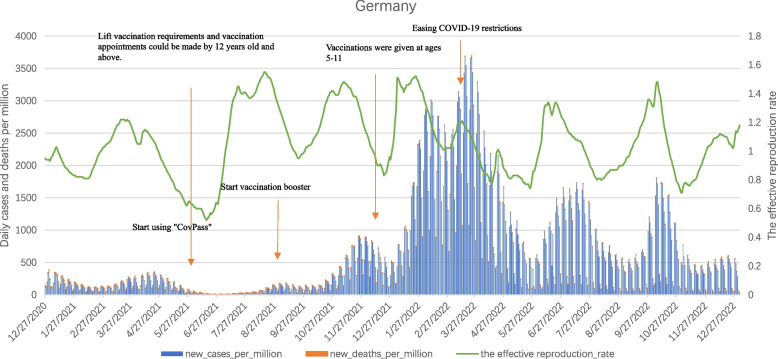
Curves of daily cases per million, daily deaths per million and the effective reproduction rate in Germany. Note1: The data were selected for the period from the start of COVID-19 vaccination in each of the three countries until 27 December 2023. Note2: The source of the data is https://ourworldindata.org/covid-vaccinations (accessed on 1 June 2023)

As can be seen from the Fig. 7, from the start of mass vaccination of COVID-19 vaccine at the end of December 2020 to the end of December 2022, the daily new cases per million curve in Germany between December 2020 and January 2021 respectively, Peaks occur from late March to May 2021, mid-October 2021 to the end of April 2022, June to July 2022, and September to October 2022. Among them, the second wave peak was significantly lower than the value of the first wave peak at the beginning of vaccination. Although the vaccination coverage rate in Germany gradually increased, with the successive epidemics of Delta and Omicron variant strains, the third epidemic peak in Germany was characterized by a long duration and a surge in the number of infections, with daily new cases per million reaching a record high of 3,693.566. In the spring of 2022, Germany relaxed its epidemic prevention restrictions, and numerous public gatherings directly led to a surge in the number of infections, with the fourth and fifth peaks.

Daily new deaths per million coincided with the peak of daily new cases per million, with the highest number of deaths in the early stages of vaccination. With the emergence and spread of the Delta and Omicron variants, the vaccine had shown a reduction in effectiveness, but daily new deaths per million were still lower than at the peak of the first wave of vaccination.

In addition, the fluctuation of Rt was similar to daily new cases per million. After the Delta variant replaced the Alpha variant in July 2021 and the Omicron variant emerged and spread in Germany at the end of November 2021, Rt suddenly increased to a higher level. And continued to fluctuate until October 2022.

Overall, the mass vaccination campaign had played a significant role in reducing infection rates and deaths before new mutated strains emerge. Due to the emergence of mutated strains and increased infectivity, Germany still experienced year-long epidemic fluctuations.

#### Denmark

**Fig. 8:**
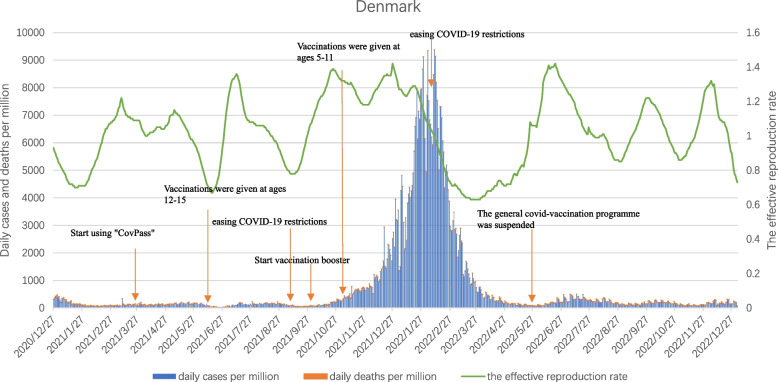
Curves of daily cases per million, daily deaths per million and the effective reproduction rate in Denmark. Note1: The data were selected for the period from the start of COVID-19 vaccination in each of the three countries until 27 December 2023. Note2: The source of the data is https://ourworldindata.org/covid-vaccinations (accessed on 1 June 2023)

As can be seen from Fig. 8, despite the high COVID-19 vaccination coverage in Denmark, with the spread of the Omicron mutant strain, daily cases per million and daily deaths per million still had an obvious short peak from December 2021 to April 2022, with the highest values reaching 9370.55 and 14.11, respectively.

In addition, Rt quickly dropped below 1 after a small spike from mid-February to mid-May 2021. After that, Rt jumped to higher levels in the time periods when new mutated strains began to spread in Denmark: July 2021, October 2021, and December 2021. Since the spring of 2022, due to the removal of COVID-19 epidemic prevention restrictions in many European countries, including Denmark, Omicron had continuously evolved subtypes with higher infectiveness and concealability, and Rt had repeatedly fluctuated in three peaks from June to December 2022.

It is not difficult to see from the development process of the COVID-19 pandemic in Denmark that even with the extensive vaccination of COVID-19 vaccine, the emergence and spread of highly infectious new variant strains still led to an increase in the number of confirmed cases and deaths. Coupled with the lifting of the COVID-19 epidemic prevention restrictions, the severity of the epidemic in Denmark had intensified.

#### Bulgaria

**Fig. 9:**
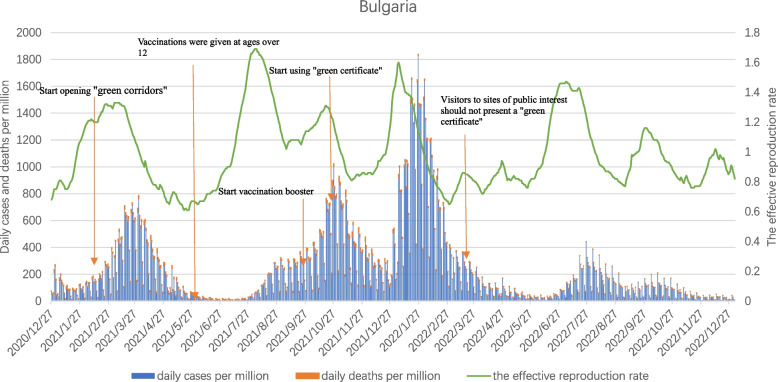
Curves of daily cases per million, daily deaths per million and the effective reproduction rate in Bulgaria. Note1: The data were selected for the period from the start of COVID-19 vaccination in each of the three countries until 27 December 2023. Note2: The source of the data is https://ourworldindata.org/covid-vaccinations (accessed on 1 June 2023)

As can be seen from Fig. 9, Bulgaria had seen four outbreak peaks from the start of vaccination on December 27, 2020 to the end of December 2022. The first peak occurred from the end of February to the end of April 2021, when Alpha strain was the mainstream. Due to the shortage of vaccine supply in Bulgaria, which had an aging population, and the low willingness of the people to vaccinate, even medical staff or doctors refused to vaccinate, insufficient immunity was formed in the population, resulting in an increase in confirmed cases. Nearly 9,000 unvaccinated older people died from COVID-19 in the first half of 2021 alone.

With the spread of the Delta variant strain, the second peak of the outbreak occurred in Bulgaria between August and December 2021, and in addition to the increase in confirmed cases, daily new deaths per million reached a record high during this phase, making Bulgaria the country with the highest number of deaths among EU member states. Although demand for vaccines in Europe is no longer in short supply, vaccination efforts in Bulgaria were still hampered by vaccine hesitancy among the population, a severe shortage of medical personnel, and a serious crisis in the health care system, with the vast majority of the population not being immunized against the virus. Even in October 2021, Bulgaria adopted "green certificates" as a mandatory condition for most indoor activities and entry into hotels and restaurants, but this measure was protested by many anti-vaxxers, and the prevention and control measures were lacking, and the domestic epidemic problem was still serious.

After a brief respite in December 2021, the outbreak in Bulgaria peaked again between January and March 2022 as the Omicron variant spread. Bulgaria, whose vaccination rates lag far behind the EU average, was affected by a new strain with very high transmissibility, with a sharp rise in daily cases per million, peaking at 1,828.234. The fourth peak occurred from July to the end of October 2022, but the value was generally low.

The fluctuation of Rt in Bulgaria is similar to the daily new cases per million, and even if Bulgaria opened the "Green corridor" in late February 2021 to start vaccinating all adults against COVID-19, it was affected by the slow vaccination process, the spread of the mutated strain, and other subjective and objective factors. Still led to a rapid increase in the number of confirmed cases and deaths.

## Discussion

Due to EU-mediated intervention during vaccine deployment, vaccination began at the same time in all three countries and COVID-19 vaccines were equitably distributed through the EU Harmonized Supply Coordination. At the same time, the use of the EU Digital Covid certificates has simplified the difficulty of travel within the EU, promoting the resumption of population movement and economic recovery within the EU. But there are country-specific differences in COVID-19 vaccine policies, specific vaccination efforts, vaccination outcomes and consequently the COVID-19 situation in the three countries, Among them, policy differences are reflected in COVID-19 vaccine authorization, recommendations and priorities for specific groups, vaccination mandates and requirements, Incentivize vaccination, and vaccine-supporting policies.

### Divergent types of responses to vaccine safety issue could fuel mistrust and hesitancy around vaccination

The COVID-19 vaccine authorized by Germany is consistent with the COVID-19 vaccine authorized by the European Commission. The only vaccines included in Denmark's general COVID-19 vaccination schedule are Pfizer/BioNTech (Comirnaty) and Moderna (Spikevax). The COVID-19 vaccine licensed by Bulgaria is part of the Danish early vaccination program, which includes Pfizer/BioNTech, Moderna, AstraZeneca and Johnson & Johnson vaccines. However, differences in the authorisation of COVID-19 vaccines also reflect different types of responses to vaccine safety issues. Due to the safety concerns of AstraZeneca and Johnson & Johnson vaccines associated with rare thrombosis, Denmark announced the withdrawal of AstraZeneca and Johnson & Johnson vaccines, while the German government once restricted AstraZeneca to people over 60 years old, only to re-authorize the vaccine for all adults a few months later. Different types of responses to vaccine safety issues may exacerbate distrust and hesitation about vaccination [18]. According to a YouGov poll published in March, 2021, more than half of people surveyed in France, Germany and Spain began to believe the Oxford/AstraZeneca COVID-19 vaccine is unsafe after several EU governments paused or limited the vaccine's use. Among Germans, 55 percent of respondents (an increase of 15 percentage points from February, 2021) believed the vaccine was unsafe [19].

### It is important to decide which groups should be prioritized for vaccination

Vaccine prioritization is an issue for countries when vaccine resources are scarce in the early stages of vaccination. The European CDC and the European Health Security Committee have prioritized elderly people (with various lower age cut‐off across countries), healthcare workers and persons with certain comorbidities [20, 21]. Observing the vaccination plans of the three countries, Bulgaria, which has a serious aging population, did not promote the priority vaccination of the elderly and high-risk groups in the early stage of vaccination, which is different from the other two countries, resulting in the death rate of COVID-19 during this period is much higher than that of the other two countries. Many studies and monitoring clearly show that the novel coronavirus is very harmful to the elderly, especially those who have chronic underlying conditions [22]. Therefore, considering the changes in the epidemic situation, the inadequate protection of the elderly and high-risk groups, and the decline in immune protection of many people over time, many countries, including Germany and Denmark, began to promote additional immune reinforcement for the elderly and high-risk groups in August 2022, vaccinate the second dose of COVID-19 vaccine booster.

### Vaccine hesitancy and mistrust in government seriously affects the promotion of vaccination

Due to the harmonization of vaccine supply in the EU, the three countries studied in this paper show equity in vaccine distribution. Bulgaria clearly lags behind Germany and Denmark in terms of speed of vaccination and coverage. The reasons for the differences in COVID-19 vaccination coverage are varied, in addition to the gap in economic and basic medical conditions and other objective factors, the degree of hesitation about vaccines among Bulgarians is evident. A survey conducted by a researcher in 2021 showed that nearly around 60% of respondents in Bulgaria were either unsure whether they would receive the COVID-19 vaccine or were certain that they would not receive the vaccine, while this figure exceeded that of the other countries surveyed by a factor of nearly two [23]. According to a survey conducted by Eurobarometer in 2021, Bulgarians had the highest level of skepticism about COVID-19 vaccines, with 26% saying they would never get vaccinated, which put them first in the EU [17]. Of the three countries studied, Bulgaria was the only one that had introduced cash incentives to increase vaccination rates among older people. Still, the results were not ideal. It is not difficult to see from the vaccination process in Bulgaria that the hesitation of the public has seriously affected the promotion of COVID-19 vaccination in the country, and the slow consumption of vaccine stocks has led to the waste of vaccines in the country.

In addition to vaccine hesitancy contributing to the disparity in COVID-19 vaccination coverage, distrust of the government is also a factor [24]. Remarkably, it has been shown that, during pandemics, citizens’ trust in public institutions has a key role [25] in determining their willingness to adopt recommended behavior. A study of public support for government responses to COVID-19 in eight Western countries, including Denmark, France, Germany, Hungary, Italy, United Kingdom, United States and Sweden, found that Denmark and Germany have the highest support [12]. In contrast, many Bulgarians were distrustful of the authorities at the start of the pandemic, as the government that led Bulgaria for most of the pandemic was plagued by corruption scandals [26].

### Safe and effective vaccines greatly reduce the probability of severe illness or death from COVID-19, and ill-prepared health systems risk plunging countries into outbreak crises

In terms of COVID-19 situation, Denmark has the highest number of Cumulative confirmed COVID-19 cases per million people, while Bulgaria has the lowest. However, Cumulative confirmed COVID-19 deaths per million people showed quite different results, with Denmark having the lowest mortality rate and Bulgaria the highest, which may be related to the high vaccination coverage in Denmark, as the COVID-19 vaccine significantly reduces the seriousness or the probability of dying from COVID-19 [27]. In addition to this, the ill-prepared health system in Bulgaria also contributed to the extraordinarily high mortality rates, as according to data published by Eurostat, Central/Eastern European countries tend to spend less as a percentage of their budgets on healthcare compared to Western countries [28]. It has been suggested that the higher COVID-19 mortality rates in CEE countries compared to Western Europe may also be due to the overall quality of healthcare [29]. The vulnerability of Eastern European countries in terms of healthcare infrastructure, medical staff, and consistent decision-making puts them at risk in an epidemic [28].

### In the face of extremely immune escape mutants, it is still important to promote global vaccination with booster shots

Observing the epidemiological curve, the successive spread of multiple mutated strains caused multiple outbreak peaks in the three countries, even as vaccination efforts continued to advance in all three countries. At present, the urgent need for vaccination booster shots is clear [30]. SARS-CoV-2 variants continue to evolve and evade, and Omicron subvariants are rapidly expanding globally, and these emerging subvariants could further compromise the efficacy of current COVID-19 vaccines and lead to a surge in breakthrough infections and re-infections [31]. In fact, although Omicron has shown significant resistance to the neutralizing activity of vaccines, convalescent serums, and most antibody therapies, COVID-19 vaccines have been shown to be effective in preventing hospitalization and severe illness even when targeted at Omicron and may reduce the risk of acute sequelae of COVID-19 (PASC or prolonged COVID) [32–34]. It has been suggested that vaccination after previous infection, or booster vaccination may increase neutralization levels and may provide severe disease protection when infected with Omicron [23, 35]. Therefore, in the face of mutant strains with strong immune escape, it is particularly important to optimize the strategy of booster vaccination and promote the vaccination of booster shots worldwide to enhance population immunity.

### Non-pharmaceutical interventions (NPIs) are essential in conjunction with vaccination

NPI refers to actions that individuals and groups can take, in addition to vaccination and drug treatment, to slow the spread of epidemics. These include measures such as testing, national and regional lockdowns, restrictions on domestic and international travel, and the closure of public places [36]. But it's worth noting that Denmark and Germany eased COVID-19 restrictions in the spring of 2022, one after another, during the Omicron epidemic. Both countries saw the increase in COVID-19 cases at the start of the transition, illustrating how relaxing national immunization programmes, even when vaccine coverage was widespread, can put more strain on healthcare systems in the short term. During this period, the widespread use of COVID-19 CERTs in EU countries had promoted cross-border travel within the EU, however, rapid population movements and uneven vaccination coverage between countries could greatly increase the likelihood of new variants emerging and lead to faster and higher peaks in new outbreaks.

### The problem of unequal vaccine distribution and vaccine waste coexist

At present, the world should continue to strengthen the vaccination of high-risk groups and key groups, which is valuable and needs to be persisted in preventing severe illness and death, and even more effectively preventing and controlling the spread of the epidemic. But in the process of vaccination, the problem of uneven distribution of vaccines and vaccine waste at the two extremes coexist. To resolve the conflict, countries need to work together to support vaccine multilateralism, Developing Countries Vaccine Manufacturers Network (DCVMN), Global Vaccine Action Plan (GVAP), International organizations such as the COVID-19 Vaccine Global Access Facility (COVAX) should adopt a coordinated and cooperative approach to inject new vitality into the response to COVID-19 in developing countries [36, 37].

## Conclusions

It is clear that despite the EU's uniform procurement and supply to ensure equitable distribution of vaccines among EU countries, there are differences in vaccination conditions and effectiveness among the three countries. What can be found is that expanding vaccination coverage has played a positive role in reducing COVID-19 infection and mortality rates and stabilizing Rt. Priority vaccination strategies targeting older people and at-risk groups have been shown to be effective in reducing COVID-19 case severity and mortality in the population. But the emergence and spread of new strains, and the relaxation of control measures, still led to multiple outbreaks peaking. In addition, vaccine hesitancy, mistrust in government and ill-prepared health systems are hampering vaccination efforts. Among the notable ones are divergent types of responses to vaccine safety issue could fuel mistrust and hesitancy around vaccination.

At present, it is necessary to promote global booster vaccination, especially for the elderly, key groups and high-risk groups [38]. Improving the fairness of vaccine distribution and reducing the degree of vaccine hesitancy are the focus of future vaccination work [39].

In general, this study compares the situation, policies and effectiveness of COVID-19 vaccination in three countries, and explores the possible reasons for the differences in policies and effectiveness among three representative countries of the European Union, and the conclusions drawn can provide a basis for national policy formulation.

## Data Availability

The datasets generated and/or analyzed during the current study are available in the [Our World in Data] repository, https://ourworldindata.org/.

## Abbreviations

COVID—19: Coronavirus disease 2019
Rt: The effective reproduction rate
